# Debate: Should Parents be able to Request Non-Resuscitation for All Extremely Premature Newborn Infants?

**DOI:** 10.1007/s41649-024-00299-0

**Published:** 2024-08-14

**Authors:** Dominic JC Wilkinson, Julian Savulescu

**Affiliations:** 1 Oxford Uehiro Centre for Practical Ethics, Faculty of Philosophy, University of Oxford, Suite 8, Littlegate House, St Ebbes St, Oxford, OX1 1PT UK; 2 John Radcliffe Hospital, Oxford, UK; 3 Murdoch Children’s Research Institute, Melbourne, Australia; 4 Centre for Biomedical Ethics, National University of Singapore Yong Loo Lin School of Medicine, Singapore, Singapore; 5 Department of Paediatrics, School of Clinical Sciences, Faculty of Medicine Nursing and Health Sciences, Monash University, Melbourne, Australia

**Keywords:** Infant, Extremely premature, Intensive care, Resuscitation, Zone of parental discretion, Parents

## Abstract

Infants who are born extremely prematurely can survive if they receive intensive medical treatment. However, they also have a high chance of dying, and a proportion of survivors have long-term health problems and disabilities. In many parts of the world, if parents request it, an extremely premature infant can receive palliative care rather than active survival-focused care at birth. But there are variations between countries as to whether or when this is permitted. To help inform ethical debates across Asia and more widely, we present two contrasting views about parental discretion and the treatment of extremely preterm infants. In questions of this nature, disagreement and dissensus are inevitable. Differences in the outcomes of treatment, the resources available, and the values of society mean that we should not expect a uniform approach. We identify points of potential consensus and compromise despite disagreement.

## Background

Approximately 0.5% of newborn infants are born extremely prematurely (i.e. before 28 weeks gestation), more than 3 months before their due date (Morgan et al. 2022). Where, in the past, almost all such infants would have died, advances in neonatal intensive care in the last 5 decades now mean that in developed countries, the majority survive. Survival is possible for infants born as early as 22 weeks gestation (four and a half months premature).

**Box 1:** Should the parents’ request for non-resuscitation be respected?

**Case X** A mother presents to a large and well-resourced maternity hospital in Singapore with premature rupture of the membranes and threatened preterm labour at 25 weeks gestation. This is her first child. Parents are professionals and are permanent residents. It has been explained that preterm delivery is possible, and they have been provided with information about the expected outcome for babies born this early. If intensive care is provided, their baby is estimated to have an 80% chance of surviving. If he survives, there would be approximately a 50% chance of him having no or mild long-term impairment and a 20% chance of major neurodevelopmental impairment (Bell, Hintz et al. 2022). On average, children born extremely preterm have an intelligence quotient 11–13 points lower than those born at full-term (Morgan et al. 2022). The parents have expressed a strong fear of baby X having a long-term disability and do not wish to take this risk. They have asked the medical team not to provide life-saving interventions but to allow the baby to die if born in the coming days.	

However, extremely preterm birth is associated with a number of serious complications including severe chronic lung disease, overwhelming sepsis, gut necrosis, and brain injury (Bell, Hintz et al. 2022). The burden and costs of treatment in the short and longer term can be considerable. For those born prior to 24 weeks, the chance of dying in the newborn period is high. As a consequence, when extremely preterm birth is anticipated, it is common for parents’ views about embarking on intensive life-prolonging therapies to be sought, at least for the most premature infants.

The range of cases where parents’ wishes about resuscitation would be sought and followed is sometimes referred to as “the grey zone” (a broader term encompassing this concept in paediatrics is the “zone of parental discretion”) (Wilkinson 2016). While there is general agreement about the existence of a grey zone for extremely premature infants, there are often ethical disagreements about the boundaries of this zone. The most common controversies relate to the lower boundary (“Lower Threshold” (Wilkinson 2016))—i.e. about how early or how small it would be appropriate to provide intensive care if parents wish this. For example, there are disagreements between and within countries about whether this threshold should be at 22, 23, or 24 weeks gestation (Wilkinson et al. 2018). However, as the above case exemplifies, there can also be questions about the upper boundary of parental discretion (the “Upper Threshold”), and how mature a preterm infant may be where it is still acceptable for health professionals to withhold life-prolonging therapies at parental request. In countries like the UK, Australia, and Canada, it is standard for active survival-focused care to be provided for infants of more than 24 weeks gestation unless there are other adverse prognostic factors present (Wilkinson 2016). In the Netherlands, the upper threshold appears to be at 26 weeks (Verweij et al. 2022). But there is relatively little ethical discussion about the basis of the Upper Threshold and how it should be determined.

Across Asia, there are few explicit guidelines relating to the thresholds for decision-making. Practice might be different between countries and from other parts of the world, in part because of differences in the outcomes of extremely premature infants. Japanese neonatal units have long experience in providing intensive care for infants as early as 22 weeks gestation and have some of the highest published survival rates in the world for such infants (Isayama 2019). (The grey zone in Japan appears to be largely from 22 to 23 weeks (Naho et al. 2018)). A guideline from Singapore published in 2021 (drawing on European and North American resuscitation guidelines), indicates that “At 24 + 0 to 24 + 6 weeks, it would be normal practice to offer full resuscitation unless the parents and clinicians make a shared decision against it based on the baby’s clinical condition and best interest principles” (Biswas et al. 2021), implying that this may be the upper boundary of the grey zone. In contrast, a guideline developed in the Philippines proposed a grey zone for resuscitation between 24 and 28 weeks (Wilkinson, Villanueva-Uy et al. 2019). This reflected data on local outcomes as well as the socio-economic challenges for parents in affording acute and long-term care. A recent paper from 40 NICUs in China (where there are no guidelines relating to decision-making for extremely preterm infants) reported that 15% of preterm infants at 25 weeks gestation had treatment withheld (“for economic or social reasons”), while this was 12.5%, 10%, and 7.4% at 26, 27, and 28 weeks gestation (Dong et al. 2023). That paper proposed that the Upper Threshold should be reduced in China from 28 to 25 weeks. But is that the right approach, and how should Asian countries determine where to draw the line?

## Debate

To help inform discussion and reflection within Asia and more broadly, we here present two contrasting viewpoints on the boundaries of parental discretion for extremely preterm infants. We will focus on the case example in order to show how the arguments are practically relevant, though the arguments will be relevant to a wider range of cases and settings.

## Pro—“Parents *should* be able to request non-resuscitation for all extremely premature newborn infants”. (Julian Savulescu)

### Non-Resuscitation of Premature Babies

As a starting point, if life-sustaining treatment can keep a human being alive, it should be employed. There is a presumption in favour of keeping human beings alive.

In medicine, there are four reasons why life-sustaining treatment should not be employed:

1.The patient validly refuses it.

2.Distributive justice requires that limited resources be used in other ways.

3.It is not in the patient’s best interests.

4.The human being lacks moral status.

In the debate around the threshold for non-resuscitation, it is arguments 3 and 4 which are commonly employed.

### Best Interests of the Child

Debates around the resuscitation of a premature infant typically concentrate on what would be in the best interests of the child. Those in favour of resuscitation argue that life will on balance be a benefit not a burden to the child. Though the child may have disabilities and suffering, these will still be compatible with a life well worth living.

In contrast, those who argue for non-resuscitation argue that the medical treatment involved (often months to a lifetime) and attendant disabilities are so severe that the child’s life would not be worth living. It is in the child’s best interests to die, often said to “die with dignity”. Life below some threshold is not worth living, while above that threshold it is worth living. In between, there is a grey zone, and we should let parents decide the fate of their child.

In philosophical terms, there are three theories of well-being: hedonistic, desire fulfilment, and objective list. According to hedonism, what is good is pleasure, and what is bad is pain. According to desire fulfilment theories, what is good is satisfying one’s desires, and what is bad is having them frustrated. On objective list theories, certain activities are good for human beings, such as developing talents, having deep and rich social relationships, having and raising children, gaining knowledge, and being creative.

Even if one could agree on which account of well-being is correct (and there is no consensus), no one has ever defined when life is not worth living on any theory, or indeed, how to measure and compare the good with the bad. Take the simplest theory—hedonism. One account of a life not worth living might be when pain is equal to pleasure or greater. But how would we measure that? It becomes even more difficult when the amount of good or bad is not certain and we have only probabilities.

Now, the problem with the best interests argument and line of a life worth living is that nobody has ever defined in a clear and operational way, let alone justified such a line. Is it, for example, a greater than 95% chance of having a future IQ of greater than 70? Is it a greater than 50% chance of normality?

I believe there is a line of a life worth living and did my doctoral thesis on it. At one end, lives which are short and full of extreme suffering, such as severe epidermolysis bullosa, seem a burden to the individual, and there is little to balance the suffering. I believe that a line should be drawn considering all three theories of well-being—hedonism, desire fulfilment, and objective list theories. A life that is clearly not worth living is one where desires cannot be fulfilled, is filled with suffering, and has little to no pleasure or worthwhile engagement with the world. But it is very difficult to justify such a line, and there is no consensus on exactly where it lies. I would draw the line very differently to others. And society appears to draw it merely related to pain and very severe cognitive impairment (near permanent unconsciousness) (Savulescu and Cameron 2019). What we are typically left with are doctors’ or judges’ own intuitions, rather than any explicit, articulated, and defensible line. But we need to draw such a line or else we consign the individual concerned to a hellish existence.

This is not to say that any choice by the parents goes. Courts rightly authorise blood transfusions of children whom Jehovah’s Witness parents refuse. However, in those cases, the child will certainly have an expected good life. It is quite different when we are considering a future life with a significant chance of significant badness.

Once a line is admitted, it is hard to give a principled argument for not respecting parents’ desires for non-resuscitation. Doctors may point out that, in the case above, “there is a 50% chance of no or mild abnormalities”.

But parents may respond “there is a 20% chance of severe abnormalities”. How are these to be weighed? Parents might also respond that the average IQ for the whole group is 88—you need an IQ of 90 to complete a tax return in the US. On average, such children are going to struggle in a modern, technologically advanced society. If we adopted a “statistically significant” approach to severe disability (that is, > 5% chance of severe disability is significant), then that would admit a 28-week threshold. Lines must be drawn but great weight should be given to parental preferences because they must rear the child and bear significant burdens themselves.

### Moral Status

Moral status refers to that interests a sentient being has and how it ought to be treated with respect to those interests. All sentient beings have an interest in not experiencing pain and suffering. This grounds animal rights. It also means human beings should not be subjected to painful procedures without good reason.

Some living beings have an interest in continued life. That is, killing them would be wrong. These are sometimes called “persons”. Fully developed human beings are persons. What makes an entity a person such that it would be wrong to kill it? The answer that the individual is a member of the species *homo sapiens* is not a satisfactory answer. Being a member of the species is related to some biological fact, such as having 46 chromosomes, but that fact is not morally relevant. (Some human beings do not, for example, have 46 chromosomes.) What is generally thought to ground personhood and moral status is some higher-order cognitive capacity, like self-consciousness (Savulescu et al. 2021b). It is wrong to kill human beings because they can conceive of themselves as existing into the future, with a biography, and desires for the future, in the way a worm cannot. It is the frustration of these desires that makes killing wrong.

If this is the ground of moral status, then some animals, such as Great Apes and dolphins, also have moral status. It would be wrong to kill them.

And moreover, some human beings lack moral status. For example, anencephalic infants will never be conscious, so they lack moral status. It is for this reason that they are allowed to die.

There is no agreement on the moral status of the fetus. Some argue it has full moral status (such as the Catholic Church), others it has no moral status (Singer 2011). and still others argue it has “some” (but it is unclear how much). These disagreements become more protracted in relation to older fetuses and late-term abortion (generally abortion after 24 weeks). Abortion after 24 weeks is allowed in several jurisdictions, such as Australia and UK. This implies it is not wrong to kill a fetus past 24 weeks, given certain criteria are met. But if it is permissible to kill a fetus at 25 weeks with an abnormality which would impact its well-being, it should be equally permissible to allow a newborn of 25 weeks to not be resuscitated for the same impact on well-being. For example, late terminations after 24 weeks have been documented for Down Syndrome (De Crespigny and Savulescu 2004). If this is justifiable, then non-resuscitation of a 25-week newborn with Down Syndrome could be seen to be equally justifiable.

Consistency with abortion, particularly late abortion, would allow for non-resuscitation of 25 weeks or older premature newborns.

I have not considered other arguments which would also support non-resuscitation. Consideration of the parent’s interests and distributive justice concerns could favour non-resuscitation, especially if parents would go on to have a child without the abnormalities associated with prematurity.

Instead, I have tried to show that “child-focused” or “patient-centred” or “best interests” arguments against non-resuscitation at 25 weeks do not succeed. Such arguments would succeed if an account of a life worth living could be provided and an account of when a human being acquires moral status were forthcoming. This is a complex issue which is not purely a medical matter. Given all these considerations, parents are perhaps in the best position to take all factors into account, though there is a role for the courts when parents are plainly making a decision which is against their child’s interests. In the absence of such clearly mistaken decision-making, we should leave parents (not doctors or courts) to decide in the “grey zone”. We could arrive at a policy through collective reflective equilibrium (we return to that below) (Savulescu et al. 2021a). But at present, we have medical or legal paternalism.

## Con –“Parents *should not* be able to request non-resuscitation for all extremely premature infants”. (Dominic Wilkinson)

Parents views should be respected for those extremely premature infants who have a very high risk of dying or of developing severe morbidity, for example, those born at 22 or 23 weeks gestation, those who are very growth restricted, or with other serious abnormalities or complications diagnosed prior to birth. However, that doesn’t apply to *all* extremely premature infants, and it doesn’t apply in the case example. Why do I think that?

1. Parents do not have a right to make (all) decisions

One reason some feel that we should allow parents to make decisions about treatment for extremely premature infants is because of the importance of parental autonomy (or ‘authority’). Parents may feel strongly (as baby X’s parents appear to) that treatment would not be right for their child. They may regard it as an infringement of their rights as parents if doctors were to refuse their request. Generally, our societies give parents significant freedom to choose how they raise their children, for example, how they are fed, clothed, and educated. That freedom reflects the importance to parents of being able to make decisions, as well as the fact that for many of those decisions, there is not a single right answer. Different families may raise their children in markedly different ways. It is not possible to say which of those ways is best. This parental authority also extends to many decisions that parents make about their children’s healthcare.

Yet parents’ freedom to make decisions for their children is importantly different from their freedom to make decisions for themselves (Bester 2021). Parents are not free to make just any decision for their child. Crucially, if parents’ decisions pose a serious risk of significant harm to the child (i.e. cross the so-called “Harm Threshold” (Diekema 2004)) those decisions should be overruled. Child protection legislation across virtually every jurisdiction recognises that it is appropriate and ethical to intervene in parental choices to protect children from harm.

It follows that parents do not have the right to refuse highly beneficial medical treatment for their child. For example, parents may not refuse a blood transfusion, or antibiotics for a serious bacterial infection, or highly effective chemotherapy (e.g. for a child with acute lymphoblastic leukaemia) (Yomiuri Shinbun 2023; Benedetti et al. 2023). That applies even if parents have strongly held religious beliefs opposing treatment (Chen 2007). This potentially applies too to life-saving, likely-to-be-effective treatment for some extremely premature infants. Parental rights and responsibilities are bounded by the best interests of the child.

2. It is in the best interests of (some) extremely premature infants to receive active treatment at birth

A different reason for withholding treatment is out of concern for the interests of the premature infants themselves. That is the major justification for not offering intensive life-prolonging therapies for most premature infants (for example, those less than 22 weeks gestation), where there is little or no prospect of the infant surviving. In such cases, the burdens of treatment outweigh the benefits, and embarking on treatment is likely to be harmful. Within the grey zone (for example, at 23 weeks gestation) there is uncertainty about the best interests of infants. Some infants will survive with no impairment. Others may survive but with variable degrees of disability. Still others will die before discharge from the hospital, sometimes after a long period of intensive care. Uncertainty (and in particular moral uncertainty) is one of the key ethical justifications for giving parents discretion about a range of decisions for their children.

However, for at least some extremely premature infants—those born at 27 or 26 weeks gestation in high resource settings (and potentially also some infants born earlier at 25 or even 24 weeks), there is no genuine moral uncertainty about whether it would be in the best interests of infants to have treatment provided. If intensive care is provided, the majority of such infants will survive, and the majority of survivors do not have severe or profound disabilities. Parents might be worried about their child having lesser degrees of disability (for example, mild or moderate learning disability or ambulant cerebral palsy), and the impact on the child’s (or parents’) life. They might prefer to have a child without impairment. However, it is very clear that the overwhelming majority of surviving ex-premature children or adults with such problems have lives that they value and that are worth living (Saigal et al. 2006).

3. It is a reasonable use of limited health care resources to provide treatment for (at least some) extremely premature infants

Finally, some may feel that the costs to society of providing neonatal intensive care to the most premature infants are too high and would justify withholding treatment.

If this argument were sound, that would provide a justification for not offering intensive care at all, rather than supporting parental wishes. However, analyses of the cost-effectiveness of providing neonatal intensive care to preterm or extremely preterm infants generally indicate that the costs of treatment fall well within the range that is usually accepted for funding in developed countries (Cheah 2019). That applies even for some of the most premature infants for whom the cost of treatment is highest due to long hospital stays. For example, in one analysis, the cost per survivor for infants born at 23 weeks gestation was US$600,000 (Yieh et al. 2022). However, because surviving infants usually survive for many years and a large proportion do not have a severe disability, the cost per life-year saved (or cost per Quality Adjusted Life Year QALY) is clearly below (within) the typical thresholds for healthcare expenditure in developed countries (e.g. $100,000/QALY). This argument for not providing intensive care is even weaker for infants born at, e.g. 25–28 weeks gestation, where the costs of treatment are lower and outcomes even better. Of course, this argument will apply differently in low-resource settings, where the threshold of affordability of treatment is lower.

However, a separate cost-related consideration relates to the direct financial impact of extremely preterm births on families. A recent systematic review identified that the prematurity-related non-medical and indirect costs to families in the first year of life of 5–20% of annual family income (King et al. 2021). If families had to share the costs of hospitalization, this was estimated to rise to 45% of household median income (King et al. 2021). This may be even more significant a factor in countries where families regularly have to bear a large part of the acute costs of care, and is cited as a significant reason for withdrawal/withholding of intensive care (Ma et al. 2019). That does not apply in the case example, since baby X’s parents will not have to pay for his acute treatment. Yet, this factor may be important in considering where countries should set the Upper Threshold.

4. Where should we draw the line?

It is very challenging to identify at what point an extremely preterm infant’s prognosis is sufficiently good that it is in their best interests to embark on treatment. There may not be a non-arbitrary way of drawing a threshold. In a UK framework relating to extremely preterm infants, a threshold of 50% chance of survival without profound impairment is used (Mactier, Bates et al. 2020). That is partly for pragmatic reasons, but it can be defended (Wilkinson 2016). In situations where there is a better than 50% chance that, if actively treated, an infant will survive without overwhelming disability, we can be confident that *most* infants actively treated will benefit from treatment. On the balance of probabilities, the benefits outweigh the harms of intensive therapy, and it is in the best interests of the infant to provide treatment. In contrast, if there is a lower than 50% chance of this, the harms at least potentially outweigh the benefits. That might mean that it is not in the best interests of infants to embark on intensive care. However, plausibly in that circumstance, there is moral uncertainty about whether to provide treatment; it would be reasonable to accede to parental requests to either provide or withhold treatment.

## Dissensus and Compromise

We have outlined above two different views about treatment decisions for extremely premature infants. It is clear that for the case example there are reasonable arguments both in favour of supporting the parent’s choice (not to resuscitate) and in favour of providing resuscitation for the infant. How then should we proceed?

In other work, we have outlined the importance of *dissensus* (Wilkinson et al. 2016; Wilkinson and Savulescu 2018). This is the idea that disagreement is inevitable in some areas of medical ethics. Such disagreement can point to the existence of moral uncertainty and should also give us some reason to be humble about our own view—acknowledging that we personally cannot be sure that we have the right answer. But it can also be possible to find common ground and compromise, notwithstanding diverging views. Here are three suggestions on which both authors agree.

1. Avoiding the horns of the dilemma

One response to dissensus and moral uncertainty should be to support individual freedom—for example, to allow patients to make choices in line with their own values—as long as that will not cause harm to others (Wilkinson et al. 2016). The challenge in paediatric decision-making is that the child will be affected and potentially harmed by the parents’ choice. Yet, there might still be a way of both respecting parents’ wishes and avoiding harm to the child. That would be to offer parents the option of adoption.

In situations where, following the birth of a child, parents are unwilling or unable to provide long-term care for the child, many societies have existing mechanisms for arranging foster care in the short-term and, in the long-term, adoption by another family (Bowie 2004). This has, in the past, been a more common practice, particularly in societies with high rates of unwanted pregnancy and limited access to termination of pregnancy (Mather 2001). The option of adoption is sometimes included within guidelines to support antenatal choice following diagnosis of fetal anomalies (for parents who do not wish to terminate, but also do not wish to care for a child with a long-term disability) (Church et al. 2020). If health professionals are convinced that it would be in the child’s best interests to survive, this option would be best for the child, while also avoiding placing a burden on the parents that they are unwilling to shoulder.

To our knowledge, there is no literature on the place or use of adoption in the context of the neonatal grey zone and parents who do not wish an extremely premature infant to receive resuscitation and active medical care. That might be because of the considerable stigma and shame associated with perceived child abandonment—such that this option is not offered, not chosen, or not reported. In some places, the availability of adoptive parents can be extremely limited. That is a particular problem for children with special needs (Miller et al. 2016), and this might apply to some extremely premature infants (though only a minority will have severe disability). If a child faces a future of considerable disruption in caregiver relationships or long-term institutional care, that could be relevant to an assessment of their best interests. However, if it is available, the option of adoption should be given to parents who do not wish for active treatment, but health professionals are not supportive of that choice.

2. Expanded choice in the setting of significant financial burden

In the case example, if there *isn’t* an option of adoption, the parents will potentially face the need to care for the child in the short and long-term. But one factor that is relevant to whether it is fair to impose this on parents relates to the anticipated financial burden. Compare a well-funded health care systems where parents do not need to pay for the child’s short term in-patient costs, nor for the child’s long-term allied health and educational support, with a low-income setting, where parents are likely to face substantial costs for the child’s medical care in both short and long-term. In the latter case, where parents face extremely high costs of medical care, and there is no option of giving the child up for adoption, that would support giving greater parental discretion for decision-making. For example, this argument was advanced in support of a wider grey zone for extremely preterm infants in a guideline developed in the Philippines (Wilkinson, Villanueva-Uy et al. 2019). On the other hand, if healthcare systems are able to offer more significant practical and financial support to parents of extremely premature infants, it may be more reasonable to insist that the child receives medical treatment.

3. Collective reflective equilibrium

We have set out arguments above in favour of supporting parental choice, and then in favour of providing resuscitation. We have also argued that other societal factors, including the availability of adoption, and the financial burden of medical and supportive care should play a role in where we set the boundaries for decisions. These arguments are important, but even with the compromise options outlined above, there is a need to determine when the outcome is sufficiently good (and not so financially burdensome) that parents should be required to accept it. There is a need to determine (at the other end of the spectrum) when the outcome is sufficiently poor that parents may not request treatment be provided. These are complicated questions, and we should not expect every community to reach the same answer about them (Wilkinson and Hayden 2018).

Elsewhere, we have described a process that combines the views of the general public along with the input of ethicists and experts (Savulescu et al. 2021a). A process of collective reflective equilibrium could potentially offer a transparent, deliberative process for a community like Singapore or other parts of Asia to reach their own answer to the debate set out in this paper.

Our aim in this paper has not been to settle the question of how we should care for extremely premature infants. It is clear that there is not a single answer to that question, and any response must be sensitive to the context of an individual country. However, we have tried to set out the key considerations that communities, professionals, and policy makers need to consider, some potential compromises that may be acceptable notwithstanding disagreement, and an important potential method for developing ethically informed policy.
